# Determination of aflatoxin M_1_ and deoxynivalenol biomarkers in infants and children urines from Bangladesh

**DOI:** 10.1007/s00204-020-02857-5

**Published:** 2020-09-03

**Authors:** Nurshad Ali, M. Manirujjaman, Sohel Rana, Gisela H. Degen

**Affiliations:** 1 Department of Biochemistry and Molecular Biology, Shahjalal University of Science and Technology, Sylhet, 3114 Bangladesh; 2 Department of Biochemistry, Gonoshasthaya Samaj Vittik Medical College, Gono University, Savar, Dhaka, 1344 Bangladesh; 3 Department of Veterinary and Animal Science, Rajshahi University, Rajshahi, 6205 Bangladesh; 4 Leibniz-Research Centre for Working Environment and Human Factors (IfADo) at the TU Dortmund, Ardeystr. 67, 44139 Dortmund, Germany

**Keywords:** Aflatoxins; deoxynivalenol; biomarkers, Infant and children, Bangladesh

## Abstract

The mycotoxins aflatoxin B_1_ (AFB_1_) and deoxynivalenol (DON) are found worldwide in crops and dietary staples. The prevalence and levels of these contaminants can vary greatly, and data in Bangladeshi food commodities are scarce. To characterize human exposure, we have conducted biomonitoring, analyzing AFM_1_ (a metabolite of AFB_1_) and DON levels in urines of adult cohorts in Bangladesh. Yet, AFM_1_ and DON occurrence has not been studied in the very young population of this country. Thus, the same methods, HPLC-FD for AFM_1_ and LC–MS/MS for DON analysis, were now applied to determine these biomarkers in urines of infants (*n* = 49) and young children (*n* = 105) in Rajshahi and Dhaka district. Overall, AFM_1_ and DON detection frequency was 43.5% and 33.4%, with 34.7% and 11.5% in infant and 47.6% and 39.4% in children urines, respectively. The mean AFM_1_ levels in all infants (9.1 ± 14.3, max 55.6 pg/mL) and children (8.8 ± 12.9, max 75.3 pg/mL) were not significantly different. The AFM_1_ mean level was slightly higher in Dhaka (9.4 ± 12.4) compared to Rajshahi (8.5 ± 13.9 pg/mL) district. The average DON level was about 2-fold higher in infant (3.8 ± 2.9, max 6.8 ng/mL) than children urines (1.6 ± 1.8, max 8.6 ng/mL), and higher in Rajshahi (2.1 ± 2.3 ng/mL) than Dhaka (1.4 ± 1.6 ng/mL) district. The biomarker-based estimated average daily DON intake (29.6 ± 108.3 ng/kg bw in infants and 36.4 ± 81.8 ng/kg bw in children) or the maximum exposure (560 ng/kg bw) do not exceed the current maximum provisional tolerable daily intake value of 1 µg/kg bw for DON, although DON exposure in infants and children is higher than that of Bangladeshi adults. The AFM_1_ urine levels in young children are somewhat lower than those found previously in adult cohorts in Bangladesh, but the frequent detection of this biomarker for AFB_1_ exposure raises further concerns, also for this vulnerable part of the population. Therefore, continuous surveillance for aflatoxins in Bangladeshi food commodities is clearly required, first to identify major sources of intake and then to reduce exposure.

## Introduction 

Aflatoxins and deoxynivalenol, secondary fungal metabolites produced by various *Aspergillus* and *Fusarium* species, are important contaminants of food commodities including dietary staples (EFSA 2017, 2020; Rushing and Selim 2019; Mishra et al. 2020). Mycotoxin exposure cannot be completely avoided, but it is essential to protect the population against acute and chronic effects. To limit exposures appropriate regulatory standards^1^ for these food contaminants are set that consider the hazardous properties of a given mycotoxin and its occurrence.

Aflatoxin B_1_ (AFB_1_), the most potent mycotoxin, exerts strong hepatotoxic and carcinogenic activity in several animal species. Exposure to aflatoxins, mainly AFB_1_, has been implicated in severe diseases in some parts of Africa and in Southeast Asia (Wild and Turner 2002; Williams et al. 2004; Groopman et al. 2008). Epidemiological studies have demonstrated a strong correlation between chronic AFB_1_ exposure and risk of developing hepatocellular carcinoma, alone or in tandem with hepatitis B virus infection (IARC 2012; Liu and Wu 2010; Sun et al. 2013). In addition, chronic exposure to aflatoxins has been linked to growth impairment (Gong et al. 2004; Turner 2013) and immune suppression in children (Turner et al. 2003). For aflatoxins known to act as mutagenic carcinogens, maximal levels are set, most strictly for infant food, and enforced in developed countries to minimize exposure of the population (van Egmond et al. 2007; Escola et al. 2019). But, in many developing countries, even when such regulation exists on paper, there are problems to achieve this goal, as described elsewhere in detail (IARC 2015).

Deoxynivalenol (DON) exposure of animals results in a number of adverse effects, including gastroenteritis, growth inhibition, and immunologic dysregulation (Pestka 2010; Alizadeh et al. 2015). Although evidence for health effects in humans related to chronic DON exposure is lacking, given the adverse effects in animals and its frequent occurrence as food contaminant, human exposure to DON is considered as a significant food safety issue (Sudakin 2003; Mishra et al. 2020). Moreover, recent food and/or biomarker-based assessments found that the mean attributed dietary exposure of children and adolescents often exceeds the tolerable daily intake of 1 µg/kg bw. set for DON and its modified forms (JECFA 2011; EFSA 2017).

The prevalence and levels of mycotoxin contamination are known to vary greatly between types of crops, regions, and season. This and variable dietary habits in different regions of the world make exposure assessments for aflatoxins and DON a rather complex task (FAO/WHO 2018; JECFA 2011). In the last decades, biological monitoring has been established as complementary approach to characterize human exposure to mycotoxins, early on for aflatoxins, then for other mycotoxins*.* The analysis of suitable biomarkers (parent compounds and/or metabolites) has a key role in investigating health concerns related to mycotoxin exposure (Turner et al. 2012), and biomarker analysis in human body fluids covers mycotoxin intake from all dietary sources and exposure by other routes (Degen 2011).

A recent review on biomarker results in human samples (blood, urine, breast milk) documents the variable patterns of mycotoxin exposure in different parts of the world (Al-Jaal et al. 2019). For the developing country Bangladesh data on contaminant levels in food commodities are scarce (Dawlatana et al. 2002; Bhuyian et al. 2013; Roy et al. 2013), and regulatory standards for aflatoxins were only recently established (BFSA 2017). In this context, biomonitoring can provide useful insights into mycotoxin exposure of the Bangladeshi population. Thus, we have conducted biomarker analysis in urines from adult residents of urban and rural areas in Raishahi and in Dhaka district: the results indicate low exposure to the *Fusarium* toxin DON (Ali et al. 2015a, b, 2016a). But AFM_1_, a biomarker of exposure to the *Aspergillus* toxin AFB_1_, has been detected in many of the urines in all adult cohorts, and at significant levels which raise health concerns (Ali et al. 2016b, 2017).

However, little is known so far about mycotoxin exposure in Bangladeshi children: two studies investigated AFB_1_ exposure in young children, one in a rural site in the North of the country, the other in an urban slum in Dhaka (Groopman et al. 2014; Mahfuz et al. 2019). Both have analyzed AFB_1_-lysin albumin in blood, a biomarker which integrates exposure during the course of several weeks. Yet, blood sampling requires medical personal whilst sampling of urine is noninvasive, easier to perform in field studies. Analysis of urinary AFM_1_ has been used in many studies as biomarker of recent AFB_1_ exposure (e.g. Polychronaki et al. 2008; Mitchell et al. 2013; Ayelign et al. 2017; Chen et al. 2018; Ezekiel et al. 2018). Urine is widely used for biomonitoring of mycotoxins which are readily excreted, for example DON: it is found in human urine as parent compound (free DON), but a far larger part are DON-glucuronides (DON-GlcA), mostly DON-15-GlcA and DON-3-GlcA (Turner et al. 2008, 2011; Brera et al. 2015). Many studies therefore apply enzymatic hydrolysis of the conjugated forms and determine’total DON’ (sum of free DON and DON-GlcA) as biomarker of exposure (e.g. Turner et al. 2008, 2010, 2011; Ali et al. 2015b, 2016a; Brera et al. 2015; Papageorgiou et al. 2018; Sarkanj et al. 2018; Wang et al. 2019). The metabolite de-epoxy-DON or DOM-1 is also found in human samples, yet less frequently, and at far lower levels than DON. Although DOM-1 is not a biomarker of exposure, an analysis of this metabolite is of some interest as indicator for detoxication of DON by the gut microbiome (Ali et al. 2016a; Wang et al. 2019).

The present study aimed to assess AFB_1_ and DON exposure in infants and children, vulnerable groups in the population, using the same methods for biomarker analysis as applied previously for adult cohorts in two districts of Bangladesh. As young children are known to ingest more food than adults on a kg body weight basis, the intake of contaminants may be higher. Moreover, as both AFB_1_ and DON may exert adverse effects on growth and immune function, co-exposure to these mycotoxins is also of interest.

## Methods

### Chemicals and reagents

Methanol (LC–MS gradient grade) was from Merck (Darmstadt, Germany). HPLC grade methanol and acetonitrile were purchased from Promochem (Wesel, Germany). Standard for AFM_1_ solution was from Sigma-Aldrich (Taufkirchen, Germany). Isotope labeled standard ([^13^C_15_] DON), deoxynivalenol (DON) and de-epoxy DON (DOM-1) were obtained from Romer Labs Diagnostics GmbH (Tulln, Austria). The β-glucuronidase/arylsulfatase from *Helix pomatia* (with specific activity 5.5 U/mL β-glucuronidase, 2.6 U/mL arylsulfatase at 37 ℃) was purchased from Roche Diagnostics (Mannheim, Germany) and used with 10-fold hydrolysis buffer (13.6 g sodium acetate hydrate, 1.0 g ascorbic acid, 0.1 g EDTA in 100 mL deionised water, adjusted to pH 5.0 with acetic acid 96%) for enzymatic pretreatment of urines. Immunoaffinity columns AflaTest^®^ WB^SR^ and DONTest™ (Vicam^®^, from Ruttmann, Hamburg, Germany) were used for sample clean-up and enrichment of the target analytes.

### Study areas and study subjects

In total, 154 urine samples were collected from Bangladeshi infants and children in Rajshahi (33 infants and 55 children) and Dhaka (16 infants and 50 children) district. Urine samples were collected between January and February 2014, a winter period in Bangladesh. In Rajshahi district, urines were obtained in rural areas (Mongol Para, Bhatpara, Habibpur and Jahubona) under Puthia Upazila. In Dhaka district, urines were obtained in rural and suburban areas (Nalam and Dhamsona) under Savar Upazila. Infants aged 1–12 months and children aged 1–6 years were included in the study if they were in good health. Demographic (age, sex) and anthropometric (height, weight) data were recorded in a brief questionnaire form. Parents or guardians were informed about the study aims and signed the consent form on behalf the participants. Urine collection containers (pots of 30 mL) and written instructions were given to the participants (parents/guardians) before the day of sample collection. On the next day, morning urine samples were collected from the participant’s house. Some urine samples for which information was incomplete were excluded from the study. The collected urine samples were first stored at −20 ℃ at the laboratory of Biochemistry Department of Gonoshasthaya Samaj Vittik Medical College, Dhaka and sent on dry ice to IfADo, Dortmund for subsequent analysis. The Institute of Biological Sciences of Rajshahi University, Bangladesh and the institutional Internal Review Board of IfADo approved the study.

### Sample preparation

Sample preparation of all urines for AFM_1_ biomarker analysis was done as described earlier (Ali et al. 2017). In brief, after centrifugation, 5 mL urine aliquots were adjusted to a pH between 5.5 and 7.0 with 1 N hydrochloric acid or 1 M sodium hydroxide. Then the urine was loaded on a AflaTest^®^ WB^SR^ column at a flow rate of 1 drop/s. The columns were washed twice with 5 mL of distilled H_2_O, then AFM_1_ was eluted (flow rate 1 drop/s) with 2 mL of methanol. Then eluates were evaporated to dryness under a stream of nitrogen at 45 ℃, and the residue was reconstituted in 250 µL of acetonitrile/water (25:75). Thus, the analyte enrichment factor was 20.

Due to limited volumes available, the remaining 120 urines (of 26 infants and 94 children) were prepared for DON biomarker analysis. Urine clean-up and enrichment of analytes were done by a slight modification of the procedure used previously (Ali et al. 2015a, b, 2016a). Briefly, 1.5 mL of each urine aliquot was hydrolyzed to cleave DON and DOM-1 conjugates by adding 125 µL of hydrolysis buffer and 20 µL of β-Gluc/ArylS enzyme and incubated overnight at 37 ℃ before sample extraction by immunoaffinity columns. Each column was rinsed with 1 mL of water and the hydrolyzed urine sample was loaded onto a DONTest™ column at a flow rate of 1 drop/sec. Then, the column was washed with 3 mL of distilled water and aglycone analytes were eluted (flow rate 1 drop/sec) from the column with 2 mL methanol. Elutes were evaporated to dryness under a stream of nitrogen at 45 ℃; the residues were dissolved in 250 µL water/methanol (90:10), vortexed and filtered through 0.45 µm pore size PTFE syringe filters before LC–MS/MS analysis. Thus, the enrichment factor was 6.

### Biomarker analysis

AFM_1_ was determined in urine by HPLC-FD following our previously established method (Ali et al. 2017) on an HPLC Shimadzu system consisting of two LC-10AS pumps, RF-10Axl fluorescence detector, SIL-10AD, Vp auto injector, CBM-20A communication module, and Shimadzu LC solution software. A C_18_ Microsorb-MV100 column (150 × 4.6 mm, 5 µm, from Agilent Technologies, Waldbronn, Germany) fitted with a C_18_ Metaguard column (10 × 4.6 mm, Microsorb A104MG) was used. The injection volume was 80 µL, and chromatographic separation was achieved by isocratic elution with mobile phase 25% acetonitrile and 75% water at a column temperature of 25 ℃ and a flow rate of 1 mL/min. The fluorescence detector was set at 360 nm excitation and 440 nm emission wavelengths; the retention time of AFM_1_ was 7.6 min. The limit of detection (LOD) was 1.7 pg/mL and limit of quantification (LOQ) was 5 pg/mL for AFM_1_. Recovery of the analyte from urine was about 90%.

Urinary levels of DON and its metabolite DOM-1 were determined by liquid chromatography with tandem mass spectrometry with a previously in-house validated method (Ali et al. 2015b, 2016a). In brief, chromatographic separation was carried out at 25 ℃ on a Nucleosil^®^ C_18_ column (100–5 material, 125 × 3 mm) with water (mobile phase A) and methanol (mobile phase B) as eluents. LC–MS/MS analysis was done on a Varian 1200-L Quadrupole MS/MS equipped with an electrospray ionization (ESI) source and a Prostar^®^ Varian HPLC system and Varian MS Workstation. DON was monitored by the transitions of m/z 295.1 → 265.1 and 295.1 → 138.1 and DOM-1 of m/z 279.1 → 248.9 and 279.1 → 231.1. The isotope labeled internal standard ([^13^C_15_] DON) was used in all quantification steps. The LODs were 0.16 ng/mL and 0.10 ng/mL for DON and DOM-1, and LOQs were 0.30 ng/mL and 0.20 ng/mL for DON and DOM-1. Recoveries for DON and DOM-1 from urine were about 92% and 85%, respectively.

### Creatinine analysis

Urinary creatinine levels were measured by a modified Jaffe method on a 96 well plate reader from TecanGenios (Blaszkewicz and Liesenhoff-Henze 2012) to account for variability in urine dilution between individual samples. Biomarker levels determined in pg/mL (for AFM1) or ng/mL (for DON) were adjusted for creatinine in urines and their concentrations expressed as pg/mg creatinine or ng/mg creatinine, respectively, to facilitate the comparison with some biomarker data published previously.

### Exposure assessment

The dietary DON intake was estimated based on results of the urinary DON analysis. The following equation was used to assess the probable daily intake (PDI) of DON among the participants

$$ {\text{PDI }}\left( {\frac{\mu g}{{kg}}{\text{Body weight}}} \right) = \frac{C \times V \times 100}{{W \times E}} $$

With *C* = biomarker concentration (DON µg/L), *V* = daily urine excretion (L), *W* = body weight (kg) and *E* = excretion rate (%). In the calculation, 24 h urinary output was assumed to be 0.5 L for children aged up to 6 years (Gong et al. 2015; Wang et al. 2019). The daily urinary DON excretion rate of 68% (Warth et al. 2013) was used, a value slightly lower than that used by others (Turner et al. 2010). The DON intake estimates (PDI) were then compared to the provisional tolerable daily intake (PMTDI) value of 1 µg/kg bw set by scientific advisory committees (JECFA 2011; EFSA 2017) to assess the risk of DON exposure.

### Statistical analysis

The software IBM SPSS version 23 was used to analyses the data. Descriptive analysis was done to determine mean, median and interquartile ranges of the analytes. Urines containing the analyte levels ≥ LOD were used in determining the mean and median values. Differences in biomarker concentrations between the infant and children cohorts, or regions were analyzed by independent sample t-test. Pearson’s correlation coefficient test (two-tailed) was applied to evaluate the correlation between biomarker levels with age and anthropometric variables. The box plot represents the distribution of central data where upper and lower limits of the box indicate 25th and 75th percentiles, respectively, and the line inside the box indicate the median value. The *p-*value lower than 0.05 is considered statistically significant.

## Results

### Characteristics of the study subjects

The basic characteristics of the study subjects are shown in Table 1. In total, 49 infants (35 males and 14 females) and 105 children (59 males and 46 females) were enrolled in the present study. The mean ages were 7.1 ± 3.7 and 37.5 ± 16.5 months for infants and children, respectively. The average height and weight were 62.4 ± 8.1 cm and 7.4 ± 2.2 kg, respectively for infants. In children, the average height and weight were 87.5 ± 13.9 cm and 12.8 ± 3.5 kg, respectively. The mean creatinine concentration in children urines (0.43 ± 0.29 g/L) was significantly (*p* < 0.01) higher than in the infant (0.13 ± 0.12 g/L) urines.

**Table 1:** Baseline characteristics of the urine donors

	All	Rajshahi	Dhaka
*N*	154	88	66
Gender of infants			
Male	35	26	9
Female	14	7	7
Gender of children			
Male	59	31	28
Female	46	24	22
Age (months)			
Infants	7.1 ± 3.7	7.1 ± 4.2	7.2 ± 2.9
Children	37.5 ± 16.5	39.3 ± 19.8	35.5 ± 11.8
Height (cm)			
Infants	62.4 ± 8.1	62.0 ± 9.0	63.1 ± 5.9
Children	87.5 ± 13.9	88.5 ± 15.3	86.3 ± 12.2
Weight (kg)			
Infants	7.4 ± 2.2	6.9 ± 2.3	8.4 ± 1.6
Children	12.8 ± 3.5	12.9 ± 4.0	12.6 ± 2.8
Creatinine (g/L)			
Infants	0.13 ± 0.12	0.14 ± 0.14	0.12 ± 0.06
Children	0.43 ± 0.29^*^	0.46 ± 0.30	0.39 ± 0.27

Value given as Mean ± SD

*p < 0.01 when compared to infant cohort. *p*-value obtained from independent sample *t* test

### AFM_1_ and DON biomarker levels in urine samples

**Fig. 1:**
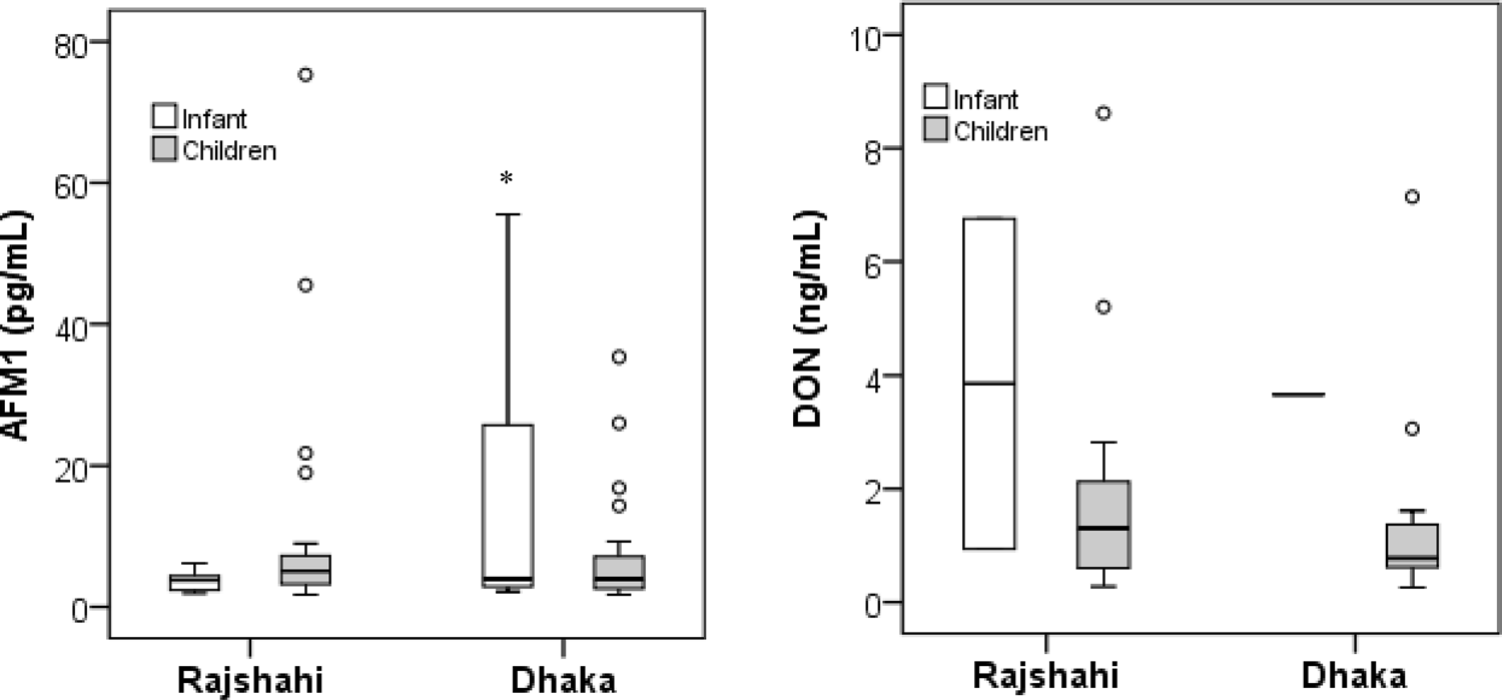
Box plots for urine levels of AFM_1_ and DON in infants and children from two regions in Bangladesh. Only positive samples (analyte ≥ LOD) are included in the graph. *p < 0.05 when AFM_1_ level in infant cohort of Dhaka district is compared to Rajshahi district. *p-*value is obtained from independent sample t-test

Results of our biomarker analysis in infants and children are given in Table 2 as non-adjusted and creatinine-adjusted urinary concentrations of AFM_1_ and DON**.** The overall AFM_1_ detection frequency was 43.5%, with 34.7% in infants and 47.6% in children urines from two regions. There was no significant difference in the mean level of AFM_1_ between all infants (9.1 ± 14.3, max 55.6 pg/mL) and children (8.8 ± 12.9, max 75.3 pg/mL) urines. When comparing region, the mean concentration of urinary AFM_1_ in all (infant and children) samples was slightly higher in Dhaka (9.4 ± 12.4) compared to Rajshahi (8.5 ± 13.9 pg/mL) district, although the difference was not significant. For infant urines, the mean AFM_1_ level was significantly higher in Dhaka (15.4 ± 19.5 pg/mL) than in Rajshahi (3.6 ± 1.4 pg/mL) district (*p* < 0.05), but not significant for children (mean 10.1 ± 15.8 in Dhaka and 7.4 ± 8.4 pg/mL in Rajshahi). Yet, the inter-individual variability of AFM_1_ in all infant and children groups is high, as depicted in Fig. 1 (left panel).

**Table 2:** Urinary levels of and AFM_1_ and DON in infants and children in Rajshahi and Dhaka district

Region	Category	AFM_1_					DON^#^				
		*N*	Positive *n*, (%)	Median (range) (pg/mL)	Mean ± SD (pg/mL)	Mean ± SD (pg/mg crea)	*N*	Positive *n*, (%)	Median (range) (ng/mL)	Mean ± SD (ng/mL)	Mean ± SD (ng/mg crea)
Rajshahi	Infants	33	9 (27.3)	3.8 (1.9–6.1)	3.6 ± 1.4	43.1 ± 40.7	22	2 (9.1)	3.8 (0.9–6.8)	3.8 ± 4.1	16.6 ± 5.9
	Children	55	27 (49.1)	5.1 (1.8–75.3)	10.1 ± 15.8	25.3 ± 29.4	49	17 (34.7)	1.3 (0.3–8.6)	1.9 ± 2.1	4.6 ± 3.9
	All	88	36 (40.9)	4.5 (1.8–75.3)	8.5 ± 13.9	29.7 ± 32.9	71	19 (26.8)	1.3 (0.3–8.6)	2.1 ± 2.3	5.8 ± 6.51
Dhaka	Infants	16	8 (50.0)	4.0 (2.2–55.6)	15.4 ± 19.5*	97.8 ± 131.4	4	1 (25.0)	3.67	3.67	27.2
	Children	50	23 (46.0)	3.9 (1.7–35.4)	7.4 ± 8.4	22.1 ± 28.8	45	20 (44.4)	0.8 (0.3–7.1)	1.3 ± 1.5	3.4 ± 2.9
	All	66	31 (47.0)	3.9 (1.7–55.6)	9.4 ± 12.4	41.6 ± 75.9	49	21 (42.9)	0.8 (0.3–7.1)	1.4 ± 1.6	4.6 ± 5.9
Both-regions	Infants	49	17 (34.7)	3.8 (1.9–55.6)	9.1 ± 14.3	68.8 ± 95.8	26	3 (11.5)	3.67 (0.9–6.8)	3.8 ± 2.9	20.1 ± 12.8
	Children	105	50 (47.6)	4.8 (1.7–75.3)	8.8 ± 12.9	23.8 ± 28.9	94	37 (39.4)	1.0 (0.3–8.6)	1.6 ± 1.8	3.9 ± 2.5
	All	154	67 (43.5)	4.4 (1.7–75.3)	8.9 ± 13.1	35.2 ± 56.9	120	40 (33.3)	1.0 (0.3–8.6)	1.7 ± 1.9	5.2 ± 6.2

Positive sample refer to urines containing the analyte ≥ LOD (LOD: 1.7 pg/mL for AFM_1_ and 0.16 ng/mL for DON). Only positive samples were considered during calculation of mean and median values

*p < 0.05 when compared to infant cohort in Rajshahi district. *p-*value obtained from independent sample t-test

^#^Due to limited urine volumes, ‘total DON’ was analyzed in 120 urines (infants = 26 and children 94) whilst AFM1 analysis included all 154 samples

The prevalence of the other mycotoxin biomarker was rather low: DON was detected in 11.5% of all infants and in 39.4% of all children urines, whilst DOM-1 was not detectable in any of the samples. The average DON concentration was about 2-fold higher in infants (3.8 ± 2.9, max 6.8 ng/mL) than in children (1.6 ± 1.8, max 8.6 ng/mL) urines. As for region comparison, the mean urinary DON level was higher in Rajshahi (2.1 ± 2.3 ng/mL) than in Dhaka (1.4 ± 1.6 ng/mL) district samples. But, differences among groups were not statistically significant, also due to considerable inter-individual variability in biomarker levels (see Fig. 1, right panel).

### Estimated dietary DON intake based on urinary analysis

The probable daily DON intake was calculated for the study subjects based on individual data of urine biomarker analysis and some additional parameters (see Methods section). In the entire study cohort, the mean daily DON intake was 34.8 ± 88.1 ng/kg bw, with 29.6 ± 108.3 ng/kg bw in infants and 36.4 ± 81.8 ng/kg bw in children in both regions (Table 3). Participants in Rajshahi district had a slightly higher calculated daily DON intake (36.5 ± 102.8 ng/kg bw) than those in Dhaka district (32.5 ± 63.0 ng/kg bw), and the highest DON intake reached 560 ng/kg bw. But, none of the subjects had an estimated DON intake that exceeds the provisional maximal tolerable daily intake (PMTDI) of 1 µg/kg bw set by scientific committees (JECFA 2011; EFSA 2017) for DON and its modified forms.

**Table 3:** Provisional daily intake (PDI) of DON (ng/kg bw)* among the cohort

Region	Category	*N*	Mean ± SD	Maximum
Rajshahi	Infants	22	27.78 ± 114.73	536.51
	Children	49	40.42 ± 98.10	559.74
	All	71	36.50 ± 102.87	559.74
Dhaka	Infants	4	36.41 ± 89.18	218.45
	Children	45	31.99 ± 60.08	340.00
	All	49	32.51 ± 63.03	340.00
Both regions	Infants	26	29.62 ± 108.28	536.51
	Children	94	36.38 ± 81.81	559.74
	All	120	34.83 ± 88.14	559.74

*Dietary DON intake was calculated based on urinary DON levels, adjusted for 24 h urine volume, assuming an 68% excretion rate and individual body weight (see methods section for details). Only positive samples were considered in PDI calculation

## Discussion

Data on the contamination of food commodities with *Aspergillus, Penicillium* and *Fusarium* mycotoxins are rather scarce for Bangladesh. To gain some insight into potential risks related to these dietary contaminants, we have previously investigated the occurrence of biomarkers of exposure to major mycotoxins in the adult population of this country (Ali et al. 2014, 2015a, 2016a, 2016b,^ 2019^; b; Gerding et al. 2015). The present study is aimed to explore the exposure of infants and young children to AFB_1_ and DON in Bangladesh. Children are considered as vulnerable group with increased susceptibility to chemicals, including mycotoxins (Makri et al. 2004; Sherif et al. 2009; Lombard et al. 2014). As outlined in the Introduction, the toxic properties of AFB_1_ and DON are quite different, the first being primarily known as potent mutagenic carcinogen, and DON (vomitoxin) for adverse effects in the gastrointestinal tract, reduced weight gain and impaired immune function. Child stunting is an emerging topic in the field of aflatoxin-related health outcomes (IARC 2015; FAO/WHO 2018; EFSA 2020); DON exposure may exacerbate this condition, independent of and along with other risk factors (Lombard et al. 2014).

The results of our biomarker analysis, the first for DON in Bangladeshi infants and children, now indicate moderate prevalence of exposure to the trichothecene mycotoxin, whilst the prevalence of AFM_1_ in their urines indicates quite frequent intake of AFB_1_ in this vulnerable part of the population (Table 2). Before discussing implications of the new results and possible sources of dietary intake, a remark is appropriate on the approach used to’translate’ biomarker data to exposure-related risks: A high renal excretion rate (68–70% within a day; Warth et al. 2013, Turner et al. 2010**)** enables biomarker-based estimates for DON exposure, and these values are then compared to the tolerable daily intake value set for this mycotoxin (see Table 3). However, only a small fraction of AFB_1_ is excreted as AFM_1_ in urine (1.5–2%; Zhu et al. 1987) which hampers a reliable back-calculation of biomarker levels to dietary AFB_1_ intake. Yet, one can compare new data on prevalence and urinary levels of AFM_1_ to results reported from other regions of the world where it served to investigate aflatoxin exposure of children.

Regarding DON biomarker analysis in urine, the detection frequency in the present study (33.3%) is close to that determined in the adult cohort in Rajshahi district (27% in summer and 31% in winter) and a pregnant women cohort in Dhaka (52%) district (Ali et al. 2015b, 2016a). But, the DON concentration in infants and children (mean 1.7, max 8.6) is higher than that found in the adults (mean 0.17, max 1.78 ng/mL in summer and mean 0.16, max 1.21 in winter) and in pregnant women (mean 0.86, max 7.16). The higher DON exposure of young children can be related to higher food consumption per kg body weight than adults and/or children preferring foods such as breads and cookies made from wheat which are more likely contaminated with DON than the typical staple food rice. Yet, no individual of the Bangladesh low age groups exceeds the tolerable daily intake value set for DON (Table 3).

It is also of interest to compare DON biomarker levels in this study to data reported in children from some other countries (Table 4)*.* Exposure to DON in Bangladesh is clearly lower than in two regions of China where 10–73% of the cohorts exceed the TDI (Wang et al. 2019), or in European cohorts: Belgium with 69% above TDI (Heyndrickx et al. 2015), Italy with 25–27.5% above TDI (De Santis et al. 2019), Norway with 20% above TDI (Brera et al. 2015), and the UK, with 33–63% above the TDI (Papageorgiou et al. 2018). In Africa, children in Cameroon have apparently lower DON exposure (Ediage et al. 2013) than those in Tanzania geometric mean 2.5 ng/mL, with 21–54% above TDI in one (Srey et al. 2014) or more in another study (Gong et al. 2015). DON has been detected now in urines of breastfed and non-exclusively breastfed infants from Nigeria (Ezekiel et al 2020). DON was also found in urines from children and adults in Haiti (Gerding et al. 2015), at levels similar to those found in Swedish children (Mitropoulou et al. 2018). DOM-1, a detoxication product of DON, was not detected in our participants, and also not found in children samples from Italy or the UK, but in some urines of Norwegian children (Brera et al. 2015).

**Table 4:** DON biomarker levels in urines from some children cohorts in different countries

Country, cohort	Positive *n* (%)	Mean (range) ng/mL	ng/mg creatinine	% exceeding TDI	Method	LOD/LOQ (ng/mL)	Reference
Bangladesh							
Infants	3/26 (11.5)	3.8 (0.9–6.8)	20.1	0	LC–MS/MS^a^	0.16/0.30	Ali et al., present study
young children	37/94 (39.3)	1.6 (0.3–8.6)	3.9	0	LC–MS/MS^a^	0.16/0.30	
Belgium, children	109/155 (70)	5.2 (0.5–32.5)	5.5	69	LC–MS/MS^b^	0.2/0.5	Heyndrickx et al. 2015
Cameroon, children	160/220 (73)	2.22^gm^	na	Na	LC–MS/MS^b^	0.04/NS	Ediage et al. 2013
China, young children*							
Henan	35/35 (100)	55.7 (max 224.1)	na	74.3	LC–MS/MS^a^	0.5/1.0	Wang et al. 2019
Sichuan	28/30 (93)	10.1 (max 56.3)	na	10.0			
Haiti, adults and children	24/142 (17)	3.2 (< LOQ–16.9)	3.6 ± na	Na	LC–MS/MS^b^	0.4/4.0	Gerding et al., 2015
Italy, children (3–9 years)							
Day 1	37/40 (93)	10.8 (1.2–138)	12.9	25	LC–MS/MS^a^	0.25/0.50	De Santis et al. 2019
Day 2	37/40 (93)	11.3 (1.4–140.9	14.9	27.5			
Nigeria, infants							
Exclusively breastfed	7/23 (30)	3.19 (0.22–19.78)	na	Na	UPLC-MS/MS^a^	0.05/0.15	Ezekiel et al. 2020
Non-exclusively breastfed	23/42 (55)	5.28 (0.23–21.34)	na	Na			
Norway children (3–9 years)	39/40 (98)	13.2 (1.6–86.9)	8.2 (0–76.1)	20	LC.MS/MS^a^	0.005/0.015	Brera et al., 2015
Sweden, children	47/50 (94)	3.9 (0.9–12.6)	na	0	LC–MS/MS^b^	NS/1.5	Mitropoulou et al. 2018
Tanzania, young children	85/166 (51)	2.5^gm^	Na	21–54	LC–MS/MS^a^	0.25/0.5	Srey et al. 2014
Tanzania, children	48/50 (96)	15.4^gm^	47.7	Na	LC–MS/MS^a^	0.25/0.5	Gong et al. 2015
UK, children (3–9 years)	40/40 (100)	29.2 (1.2–141)	41.6 (5.3–219.0)	33–63	LC–MS(MS^a^	0.12/0.25	Papageorgiou et al. 2018

*na* not available, *NS* not stated, *gm* geometric mean

*age group 1–6 years, 3 urines per child collected on 3 consecutive days

^a^Immunoaffinity column clean up and tailored method

^b^Multi-biomarker method

These biomonitoring studies (Table 4) show a wide range of DON exposures in the pediatric population of different countries, but also differences between regions of a country, as in China (Wang et al. 2019). The extent of DON exposure can be explained in part by differences in dietary habits: food items such as wheat bread, pasta, breakfast cereals, bran rolled flakes and baked goods are major sources of DON exposure in European populations (Brera et al. 2015; EFSA 2017), but these foods are far less often consumed in Bangladesh. Biomarker levels not only differ between countries, but also between years and season (Gratz et al. 2014; Ali et al. 2016a). This reflects the quite variable DON contamination of many crops worldwide (Mishra et al. 2020). Thus, one should keep in mind that biomonitoring sheds a light on the DON exposure in a given cohort and sampling season, and should be followed up, in particular when data indicate exceedance of TDI-values in vulnerable groups (Papageorgiou et al. 2018).

Regarding AFM_1_ analysis, the biomarker has been frequently detected (43.5% in the entire cohort), with 34.7% in infants and 47.6% in children urines from two regions (Table 2), and with a high inter-individual variability in AFM_1_ levels of all groups (Fig. 1). The new results are first compared with data in Bangladeshi adults and then with findings for children of other countries. The AFM_1_ urine concentrations in infants (mean 9.1, max 55.6 pg/mL) and children (mean 8.8, max 75.3 pg/mL) are somewhat lower than those measured in the adult (13.5 pg/mL, max 104 pg/mL in summer and 27.7 pg/mL, max 189.9 pg/mL in winter) and pregnant women (13.9 pg/mL, max 141.5 pg/mL) cohorts of this country (Ali et al. 2017). These biomarker data suggest that foods consumed by adults and by children contain notable levels of aflatoxin B_1_, whilst the sources of mycotoxin intake may differ between both groups.

Urine AFM_1_ levels in young Bangladeshi children are far lower than levels found in children of some African countries (Table 5), i.e. in Cameroon (Ediage et al. 2013), Guinea (Polychronaki et al. 2008), Nigeria (Ezekiel et al. 2020, 2018; Sarkanj et al. 2018), Sierra Leone (Jonsyn-Ellis 2001) and Tanzania (Chen et al. 2018). However, the AFM_1_ mean level in our cohort is higher than that reported in children urines in Egypt (Polychronaki et al. 2008), and it approaches levels found in Ethiopia (Ayelign et al. 2017). Also, the average Bangladeshi values are in a similar range as those found in children in Colombia, South America (Sanchez and Diaz 2019). The children data (Table 5), and a previous overview of AFM_1_ biomarker data for adult cohorts of different continents and countries (Ali et al. 2017) illustrate the wide range of exposure to AFB_1_. Again, exposure reflects different dietary habits in these populations and also different degrees of aflatoxin contamination in the crops and foods locally produced and consumed. For instance, in Africa maize and groundnuts continue to be the main sources of aflatoxin exposure (Gong et al. 2018; Xu et al. 2018).

**Table 5:** AFM_1_ level in urines from some children cohorts in different countries

Country, cohort	Positive *n* (%)	Mean (range) pg/mL	pg/mg creatinine	Method	LOD/LOQ (pg/mL)	Reference
Bangladesh, children						
Infants	17/49 (35)	9.1 (1.9–55.6)	68.8	HPLC-FD^a^	1.7/5.0	Ali et al., present study
young children	50/107 (48)	8.8 (1.7–75.3)	23.8			
Cameroon, children	31/220 (14)	330^gm^ (< 10–4700)	na	LC–MS/MS^b^	10/20	Ediage et al. (2013)
Colombia, children	40/96 (42)	16 (LOD-48.5)	na	HPLC-FD^a^	2/6	Sanchez & Diaz (2019)
Egypt, children	4/50 (8)	5.5 (5.0–6.2)	na	HPLC-FD^a^	5/-	Polychronaki et al. (2008)
Ethiopia, children	14/200 (7)	64 (63–70)	na	LC–MS/MS	25/50	Ayelign et al. (2017)
Guinea, children	32/50 (64)	97 (8.0–801)	na	HPLC-FD^a^	5/-	Polychronaki et al. (2008)
Haiti, adults and children						
Port-au-prince	20/147 (14)	Na	43.7 (3.97–202)	HPLC-FD^a^	4/10	Schwartzbord et al., (2016)
Quartier morin	48/219 (22)	Na	116 (2.44–775)			
Italy, adults and children	3/52 (6)	68 (20–146)	na	LC–MS/MS^b^	NS/20	Solfrizzo et al. (2013)
Nigeria, children (2–7 years)	13/13 (100)	280 (110–510)	na	ELISA	6/-	Ezekiel et al. (2018 )
Nigeria, adults, adolescents and children	17/120 (14)	300 (LOD–1500)	na	LC–MS/MS^b^	50/150	Ezekiel et al. (2014)
Nigeria urines (reanalysis)	87/120 (72.5)	40 (1–620)	na	UPLC-MS/MS^c^	–/1	Sarkanj et al. (2018)
Nigeria, infants						
Exclusively breastfed	11/23 (4)	23 (23)	na	UPLC-MS/MS^a^	0.0003/0.001	Ezekiel et al. (2020)
Non-exclusively breastfed	55/42 (12)	166 (32–504)	na			
Sierra Leone, children						
Dry season	104/244 (43)	na (500–374,000)	na	HPLC	5–50	Jonsyn-Ellis (2001)
Rainy season	97/190 (51)	na (100–124,000)	na			
Tanzania (6–14 months) in 3 villages; repeated visits	72/84 (86%)	36.5^gm^ (15–2840)	na	ELISA	10–15	Chen et al. (2018)

*na* not available, *NS* not stated, *gm*geometric mean

^a^Immunoaffinity column clean up and tailored method

^b^Multi-biomarker method ^c^multi-biomarker method with enzymatic hydrolysis and sample clean-up by SPE

So far there are only two studies on aflatoxin occurrence in food commodities in Bangladesh. Roy et al. (2013) analyzed AFB_1_ in rice, lentils, wheat flour, dates, betelnut, red chilli powder, ginger and groundnuts: mean levels in 5 of these 8 commodities were above EU regulatory limits, and the highest levels were found in dates and groundnuts. Bhuiyan et al. (2013) analyzed total aflatoxins in maize, rice and wheat samples collected in all districts of Bangladesh at six times during a year: the highest incidence and level of contamination was found in maize; the incidence and contaminant level were lower, yet still significant in rice and in wheat, all showing considerable seasonal variability. Both datasets have been used by others to calculate total aflatoxin levels in various food commodities in Bangladesh (Table 1 in Saha Turna and Wu 2019). Then, considering also average dietary consumption data for each of these items, they assessed aflatoxin exposure by dates, groundnuts, lentils, chili/spices, wheat, maize and rice (Table 2 in Saha Turna and Wu 2019). The highest contribution to total aflatoxin exposure was from rice. Rice is the main dietary staple in Bangladesh, and often consumed with curries prepared with several spices. Previously, we noted higher urine AFM_1_ levels in adult people who consume more rice per day (Ali et al. 2017) which points also to rice as one important source of AFB_1_ intake (Ali 2019). In their paper Saha Turna and Wu (2019) comment also on the high AFB_1_ levels in dates, which are consumed mainly during Ramadan in Bangladesh and other Muslim countries. We suggest that along with rice, wheat-based bakery products, also dates may be a relevant source of AFB_1_ intake as young children prefer sweet types of food. Furthermore, a recent screening of cow milk and milk products by ELISA reveals frequent occurrence of AFM_1_ (Ali et al. unpublished results). As young children are fond of milk and milk based products, this may have also contributed to AFM_1_ exposure in our cohort.

AFB_1_ exposure has been investigated before in another region of Bangladesh (a rural area in Rangpur) by means of the AFB_1_-lysine albumin adduct analysis in blood samples of pregnant women and later on in their 2-year-old children (Groopman et al. 2014). Median levels of this biomarker were 25.35 and 18.08 pg AFB_1_-Lys/mg albumin in the first and third trimester, respectively, and 13.79 pg AFB_1_-Lys/mg albumin in the children. These results, discussed by the authors in the context of biomarker data for cohorts in other countries, document rather high AFB_1_ exposures of their cohort in the Northwest of Bangladesh between 2008 and 2012. A recent longitudinal study in an urban slum in Dhaka city assessed AFB_1_ exposure of children at the age of 7, 15, 24 and 36 months and reported a geometric mean of 1.07 pg AFB_1_-Lys/mg albumin and a range of 0.04–123.5 pg AFB1-Lys/mg albumin (Mahfuz et al. 2019). In this study, a reduction in breastfeeding prevalence, with concomitant introduction of family food, corresponded with an increase in AFB_1_-lysin adduct detection at 36 months, and 62% of the children were exposed at the end of their 3^rd^ year of life. Of interest is also the seasonal variation in AFB_1_ biomarker prevalence, with the highest detection observed during and after the monsoon period which provides optimal conditions for fungal growth and aflatoxin contamination (Mahfuz et al. 2019).

Overall, the results of our study with determination of AFM_1_ metabolite in urine, and the two studies (Groopman et al. 2014; Mahfuz et al. 2019) that measured AFB_1_-lysin albumin adduct in blood plasma, document widespread exposure of young children in several parts of Bangladesh. This and previous data on frequent AFB_1_ exposure of adult and pregnant women cohorts (Ali et al. 2017; Groopman et al. 2014) raise concern with regard to dietary intake of this carcinogenic mycotoxin in the Bangladeshi population. Further efforts to analyse aflatoxin contamination are clearly needed to identify major sources of aflatoxin intake, and establish surveillance in food commodities with the aim to protect the population against long-term adverse health effects. Bangladesh has issued in mid 2017 regulation for aflatoxin contamination of certain food items, namely groundnuts, almonds, Brazil nuts, hazelnuts, pistachios and AFM_1_ in milk (BFSA 2017). Yet, a recent risk assessment of aflatoxin-related liver cancer in Bangladesh concluded that the new regulations are unlikely to significantly reduce the risk of this cancer in the country (Saha Turna and Wu 2019). Indeed, considering food contaminant data available (vide supra), cereal-based commodities including rice, as well as pulses, spices and other items are likely to contribute far more to overall AFB_1_ exposure than various types of nuts. Thus, we recommend to conduct regular surveys on aflatoxin contamination, at least in major staples (Ali 2019), and consider also further biomonitoring as this integrates human exposure from all sources.

## Conclusion

This study applied sensitive biomonitoring methods to assess for the first time aflatoxin and DON exposure among infant and children cohorts in Bangladesh. DON exposure appears to be of low concern, with intake estimates below tolerable levels. But, the prevalence and levels of AFM_1_ in infant and children urines indicate widespread contamination of the children’s diets with the carcinogenic mycotoxin AFB_1_, a finding which raises serious health concerns for this vulnerable population. Continuous surveillance of aflatoxins in Bangladeshi food commodities are urgently needed, in order to identify major sources of intake, and then take appropriate steps to further reduce risks from exposure-related adverse health effects.

## Footnotes

1 Scientific bodies evaluate dose–response relationships for the most critical effect observed in animal studies. Then considering also mode of action they derive tolerable daily intake values by a margin of safety approach (e.g. for DON) whereas a margin of exposure approach is used for mutagenic carcinogens like aflatoxins (details in FAO/WHO 2018; JECFA 2011; EFSA 2017; EFSA 2020).
